# Attenuation of *Staphylococcus aureus* Biofilms and Virulence by 3-Fluorocatechol

**DOI:** 10.3390/antibiotics14121240

**Published:** 2025-12-08

**Authors:** Taehyeong Kim, Nazia Tabassum, Aqib Javaid, Fazlurrahman Khan

**Affiliations:** 1 Department of Applied Biosciences, Kyungpook National University, Daegu 41566, Republic of Korea; taykim0105@knu.ac.kr; 2 Marine Integrated Biomedical Technology Center, The National Key Research Institutes in Universities, Pukyong National University, Busan 48513, Republic of Korea; nazia99@pukyong.ac.kr; 3 Research Center for Marine Integrated Bionics Technology, Pukyong National University, Busan 48513, Republic of Korea; 4 Interdisciplinary Program of Marine and Fisheries Sciences and Convergent Technology, Pukyong National University, Busan 48513, Republic of Korea; aqibj@pukyong.ac.kr; 5 Ocean and Fisheries Development International Cooperation Institute, Pukyong National University, Busan 48513, Republic of Korea; 6 International Graduate Program of Fisheries Science, Pukyong National University, Busan 48513, Republic of Korea

**Keywords:** *Staphylococcus aureus*, 3-fluorocatechol, antibiofilm, antivirulence, molecular docking

## Abstract

**Background/Objectives:** *Staphylococcus aureus* is a well-known opportunistic pathogen that causes a wide range of infections, from cutaneous blemishes to potentially fatal systemic diseases. The increasing prevalence of antibiotic-resistant bacteria highlights the critical need for alternative therapeutic methods that target virulence factors rather than growth. **Methods:** The antibacterial activity of 3-fluorocatechol (3-FC) against bacterial and fungal pathogens (e.g., *Candida albicans*) was determined by broth microdilution to establish the lowest inhibitory concentration. The antibiofilm impact of 3-FC against *S. aureus* was evaluated using crystal violet staining and viable colony counts, followed by scanning electron microscopy to visualize the biofilm architecture. The methanol extraction method was used to quantify staphyloxanthin synthesis in *S. aureus* cells. Furthermore, in silico molecular docking was used to evaluate 3-FC binding interactions and provide mechanistic insight into its impacts on *S. aureus* biofilms and virulence-associated factors. **Results:** Although the study showed that 3-FC exhibits weak antibacterial activity against *S. aureus* (MIC > 2048 µg/mL), it shows effective inhibition of up to 86.5% at sub-inhibitory doses during the initial stage of biofilm formation. The CFU enumeration also confirms the significant reduction of viable cell count of *S. aureus* in the presence of sub-MIC of 3-FC. The SEM analysis confirms disruption of the *S. aureus* biofilm architecture in the presence of a sub-MIC of 3-FC. Furthermore, the eradication of mature *S. aureus* biofilm at a sub-MIC dose of 3-FC was 60.6%. 3-FC significantly reduced staphyloxanthin formation, a vital antioxidant pigment that contributes to bacterial pathogenicity, with a maximal suppression of 66.3% at 2048 µg/mL. Molecular docking analyses provide further insight into the molecular basis of 3-FC activity, revealing strong binding affinities with numerous *S. aureus* virulence regulators and enzymes, suggesting interference with quorum-sensing, adhesion, and oxidative-stress response pathways. **Conclusions:** Collectively, our findings indicate that 3-FC has antibiofilm and antivirulence properties against *S. aureus.* Furthermore, this study suggests 3-FC as a viable structural scaffold for the development of a novel anti-infective agent to treat chronic staphylococcal infections.

## 1. Introduction

*Staphylococcus aureus* remains a formidable bacterial pathogen, causing infections that range from superficial skin lesions to life-threatening systemic diseases, including bacteremia, endocarditis, and pneumonia [1]. The pathogen’s success is attributed to its extensive virulence arsenal, including surface adhesins, secreted toxins, and biofilm-forming ability [1]. Methicillin-resistant *S. aureus* (MRSA) emerged in hospitals by the late 1960s and unexpectedly spread to communities in the 1990s, becoming prevalent worldwide [2,3]. *Staphylococcus aureus* biofilms represent a critical pathogenic mechanism enabling bacterial persistence and antibiotic resistance. These structured multicellular communities are embedded within a self-produced extracellular polymeric matrix composed of polysaccharides, proteins, and extracellular DNA [4]. Biofilm formation occurs through distinct stages: initial attachment to surfaces, proliferation into microcolonies, matrix production, and eventual dispersal [4,5]. This phenotypic characteristic enables *S. aureus* to colonize surfaces (both biotic and abiotic), including medical devices such as implants and catheters, making it a leading cause of device-associated infections [6,7]. The EPS matrix of the biofilm restricts antibiotic penetration, supports reduced metabolic activity, and facilitates horizontal gene transfer/or mutations, all of which contribute to the development of antimicrobial resistance and chronic infections [8,9,10]. This resistance mechanism significantly complicates clinical treatment and has led to an increased focus on developing novel anti-biofilm therapeutic strategies [11]. Targeting biofilm formation and associated virulence mechanisms, such as adhesion, quorum sensing, and toxin production, has emerged as a promising therapeutic strategy [12,13].

A recent study found that halogenated catechols, catechol-based chemicals modified with halogen atoms such as chlorine, bromine, fluorine, or iodine, exhibit antibacterial activity through interactions with bacterial dioxygenases. Several reports showed that halogenated catechols can act as suicide substrates for catechol dioxygenases, leading to enzyme deactivation and disruption of the aromatic biosynthetic pathway in bacteria [14,15]. The mechanism involves the high electronegativity of halogen atoms, which destabilize chemical intermediates and cause irreversible enzyme damage [16]. According to studies, halogenated dopamine derivatives integrated into polymers and coatings kill more than 99% of Gram-positive bacteria [14]. Structural studies show that halogenated catechols bind to the active site of dioxygenase in specific orientations, with distinct binding modes observed for differently substituted catechols [17]. The discovery of the specific inhibitory effect of intradiol and extradiol catechol dioxygenases has validated this strategy for antibacterial applications [18]. These findings support the use of fluorinated catechols as potent antibacterial drugs targeting critical metabolic pathways. It is reported that the fluorinated compounds have distinctive features that make them useful for mechanism-based enzyme inhibition. The high electronegativity property of fluorine makes it easier to remove fluoride from metabolic intermediates, allowing for the creation of suicide substrates that cause irreversible inactivation of metabolic enzymes [19].

Additionally, a number of studies have demonstrated that fluorinated compounds exhibit superior antibacterial and antibiofilm activity compared to their non-fluorinated counterparts with the same properties. A fluorinated bisindole alkaloid showed increased biofilm-eradication activity, leading to a 256-fold reduction in the oxacillin minimum inhibitory concentration (MIC) against MRSA [20]. It is also evident that the fluorinated pyrrolomycins showed substantial anti-staphylococcal biofilm activity, good pharmacokinetic profiles, and no bacterial resistance [21]. Similarly, a fluoroaryl-bichalcophene derivative, specifically MA-1156, demonstrated robust antibacterial and antibiofilm activity, with a minimum inhibitory concentration (MIC) of 16 µM [22]. Additionally, fluorinated polymer micelles enhanced ciprofloxacin delivery and showed superior antimicrobial activity against MRSA biofilms [23]. These findings support the potential of fluorinated compounds as effective antibiofilm agents; however, specific studies on 3-fluorocatechol (3-FC) against *S. aureus* are not currently available in the literature. Earlier, it was reported that 3-FC causes oxygen-dependent, irreversible inactivation of catechol 2,3-dioxygenases through the oxidation of the active site Fe(II) to Fe(III), with a rate constant of 2.38 × 10^−3^ s^−1^ [24]. Similarly, 4-methylcatechol acts as a suicide inhibitor by promoting Fe(II) oxidation [25].

The present study aimed to investigate the antimicrobial potential of 3-FC against Gram-positive and Gram-negative bacterial pathogens and the fungal pathogen *Candida albicans*. The primary objective of this study is to thoroughly examine the antibiofilm and antivirulence potential of 3-FC against *S. aureus*. These phenotypic inhibitory activities were investigated through the mechanistic action of 3-FC using in silico molecular docking against biofilm and virulence-associated factors of *S. aureus*.

## 2. Results and Discussion

### 2.1. Antimicrobial Role of 3-FC Towards Pathogens

The global rise of antibiotic-resistant *S. aureus*, particularly MRSA strains, has necessitated the development of alternative therapeutic strategies that target virulence factors rather than bacterial growth to minimize the selection pressure for resistance [26,27]. Biofilm development is an important virulence factor, accounting for more than 80% of bacterial infections and contributing considerably to antibiotic resistance and chronic diseases [28,29]. Antivirulence techniques based on small-molecule inhibitors have shown promise, with drugs such as diflunisal analogues inhibiting hemolysis, proteolysis, and biofilm formation without affecting bacterial growth [30]. Reports indicated that fluorinated compounds offer unique advantages for inhibitor design due to fluorine’s electronegativity and leaving-group ability [31]. Studies on suicide inhibition of tyrosinase demonstrate that catechol derivatives can act as suicide substrates, with 3,6-difluorocatechol showing distinct oxidation behavior that prevents suicide inactivation [16,32]. The inhibition mechanisms of fluoroquinolone antibiotics on CYP3A4 have been reported to involve the formation of a metabolite–intermediate complex [33]. Polymers based on catechol have been shown to exhibit antimicrobial activity through various mechanisms, including the generation of reactive oxygen species (ROS) and the complexation of metal ions [34].

The antibacterial activity of 3-FC against *S. aureus* was examined by measuring its MIC, which was found not to completely stop growth even at the highest concentration tested (2048 µg/mL), showing a MIC of >2048 µg/mL (Table 1). The half-maximal inhibitory concentration (IC_50_) was determined to be 1604 µg/mL using nonlinear regression analysis. Table 1 summarizes 3-FC’s antibacterial activity against different bacterial strains. *P. aeruginosa* and *C. albicans* had the lowest MIC values, both measured at 512 µg/mL.

Research on halogenated compounds and catechol derivatives demonstrates varying antimicrobial activities against *S. aureus* and *P. aeruginosa*. Synthesized flavonoids with halogen substitutions displayed notable activity against *S. aureus* (MIC = 31.25–125 μg/mL), with chalcones proving most effective [35]. Similarly, halogenated biotin protein ligase inhibitors promise results, with 5-fluoro-1,2,3-triazole achieving an MIC of 8 μg/mL against *S. aureus* [36]. Natural catechin compounds exhibited moderate activity, with (+)-catechin showing MICs of 600 μg/mL against both *S. aureus* and *P. aeruginosa* [37]. Green tea catechins demonstrated MICs of 62.5–250 μg/mL against bacterial strains and showed synergistic effects when combined with gentamicin [38]. Green tea extract alone required higher concentrations, with MICs of 400 μg/mL against *S. aureus* and 800 μg/mL against *P. aeruginosa* [39]. Oxazolidinone–catechol conjugates showed enhanced activity against *P. aeruginosa* (218–1024 μM) compared to linezolid [40]. These findings suggest that halogenation and structural modifications can significantly improve antimicrobial potency. The antimicrobial mechanism appears to involve the generation of reactive oxygen species and the induction of oxidative stress [37,41]. It has been reported that the fluorinated and halogenated substituents significantly enhance the biological activity and drug-like properties of small molecules through multiple mechanisms. Trifluoromethyl (CF_3_) and trifluoromethoxy (OCF_3_) groups improve lipophilicity, membrane permeability, and metabolic stability while maintaining favorable ADME properties [42,43]. These modifications have demonstrated superior antimicrobial efficacy, with OCF_3_-substituted chalcones showing greater activity than CF_3_ analogs against both Gram-positive and Gram-negative bacteria [44]. Several studies suggest that combining catechol compounds with conventional antibiotics can produce synergistic effects [38,45].

The inhibitory activity of 3-FC against *Candida albicans* is a significant finding, as it demonstrates that its effects extend beyond bacteria, positioning it as a broad-spectrum antimicrobial agent. The suggested antibacterial mechanism for halogenated catechols is the suicide inhibition of catechol dioxygenases. However, as a fungus, *C. albicans* lacks this enzyme. This finding implies that 3-FC must use a different mechanism to prevent fungal growth. This finding is consistent with prior studies on catechol against *C. albicans* [46]. This study reported that catechol exhibited an MIC of 1024 μg/mL against *C. albicans* and acted as an anti-virulence agent, inhibiting key pathogenic traits, including biofilm formation and the Ras-cAMP-PKA signaling pathway. Given the structural similarity between catechol and 3-FC, it is plausible that 3-FC shares this mechanism, possibly enhanced by the fluorine substituent.

### 2.2. Biofilm Inhibitory Role of 3-FC

**Figure 1:**
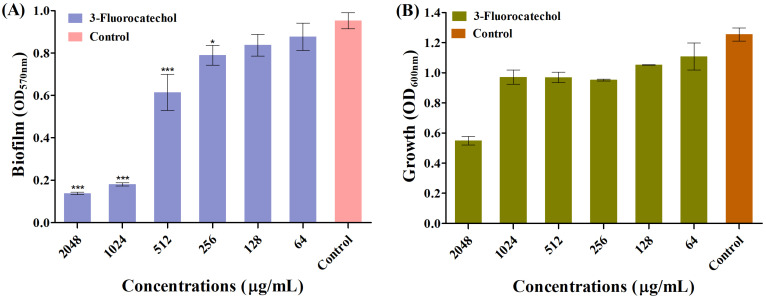
(**A**) Inhibition of *S. aureus* biofilm in the presence of 3-FC ranging from 64 to 2048 µg/mL and (**B**) Cell growth of *S. aureus* in the presence of the sub-MIC of 3-FC. The control represents the untreated group. The biofilm inhibitory effect was quantified using the crystal violet staining method at 570 nm. Asterisks indicate a statistically significant difference compared to the untreated control (* *p* < 0.05, *** *p* < 0.0001).

**Figure 2:**
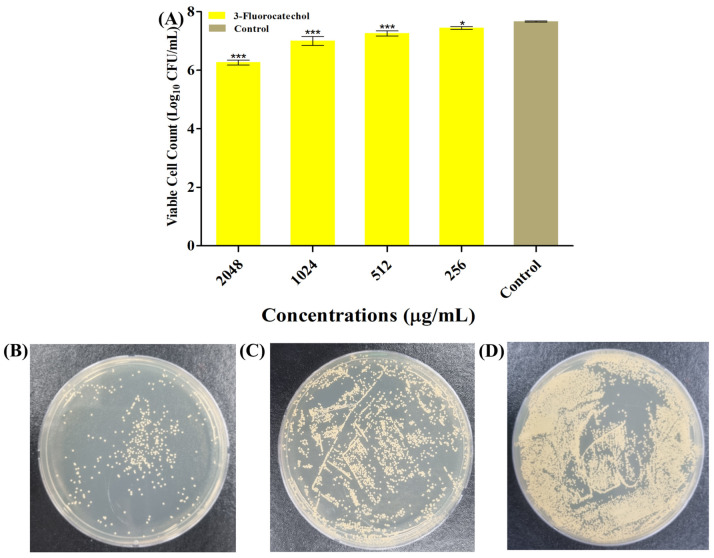
(**A**) Viable cell counts were determined by the colony-forming unit (CFU) enumeration. The results were expressed as Log_10_ CFU/mL, (**B**) TSA plate treated with 2048 µg/mL of 3-FC, (**C**) TSA plate treated with 1024 µg/mL of 3-FC, and (**D**) TSA plate without 3-FC treatment. Asterisks indicate a statistically significant difference compared to the untreated control (* *p* < 0.05, *** *p* < 0.0001).

The effect of 3-FC on biofilm formation was investigated at sub-inhibitory concentrations (sub-MICs). The inhibition of biofilm of *S. aureus* in the presence of the sub-MIC of 3-FC was found to be in a concentration-dependent manner (Figure 1B). Reductions of 82.2% and 86.5% were observed at 1024 µg/mL and 2048 µg/mL, respectively. At these treated sub-MICs of 3-FC, there was no cell growth inhibition, suggesting that the cells were freely available to form biofilm (Figure 1A). To further validate the biofilm inhibition properties of 3-FC, a colony-forming unit (CFU) enumeration was performed (Figure 2), and the results showed that treatment with 2048 µg/mL of 3-FC resulted in an average 1.4 Log_10_ CFU/mL reduction in viable cells as compared to the control (Figure 2A). Halogenated compounds exhibit potent anti-virulence effects against *S. aureus*, with fluorine and chlorine substitutions modulating hemolysis, proteolysis, and biofilm formation through distinct pharmacophores [30]. Fluorinated phenazines and acridines eradicate MRSA biofilms by inducing iron starvation [47], while halogenated catechols demonstrate over 99% killing efficiency against Gram-positive bacteria [14].

**Figure 3:**
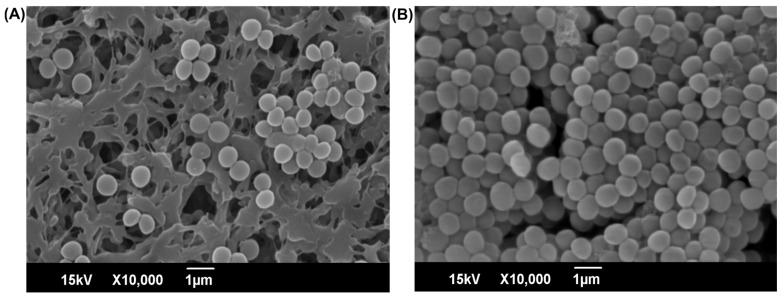
SEM images show the structural changes in *S. aureus* biofilms after treatment. (**A**) In the 3-FC-treated sample, a significant reduction in bacterial cell density was observed, and (**B**) the untreated control sample exhibited well-formed cellular aggregates.

The biofilm structure was visualized by scanning electron microscopy (SEM) at a concentration of 2048 µg/mL, which showed the maximum inhibition (Figure 3). The sample treated with 2048 µg/mL of 3-FC (Figure 3A) showed reduced cell adherence on the nylon membrane surface and biofilm development. In contrast, the untreated control sample exhibited a thick biofilm cell architecture (Figure 3B). Previous studies on *S. aureus* biofilm suppression have identified potential natural and synthetic substances with concentration-dependent effects. Flavonoids exhibit antibiofilm activity, with aglycone forms such as myricetin, hesperetin, and phloretin showing >70% biofilm inhibition at sub-MIC concentrations (1–256 μg/mL) against *S. aureus* strains. This is consistent with the sub-MIC inhibitory potential of 3-FC [48]. In particular, phloretin exhibits dose-dependent biofilm inhibition at 0.5 × MIC in multiple strains [49]. Natural compounds, including salicylaldehyde, vanillin, and cinnamaldehyde derivatives, achieve a 15–92% reduction in biofilm at concentrations of 1–10 mg/mL [50]. Marine-derived bisindole alkaloids exhibit both antimicrobial and anti-biofilm properties, with fluorinated analogues demonstrating enhanced activity against preformed biofilms [20]. These findings highlight the potential for developing novel anti-biofilm therapeutics targeting drug-resistant *S. aureus* infections [13].

### 2.3. 3-FC Eradicates the Preformed Mature Biofilm of S. aureus

**Figure 4:**
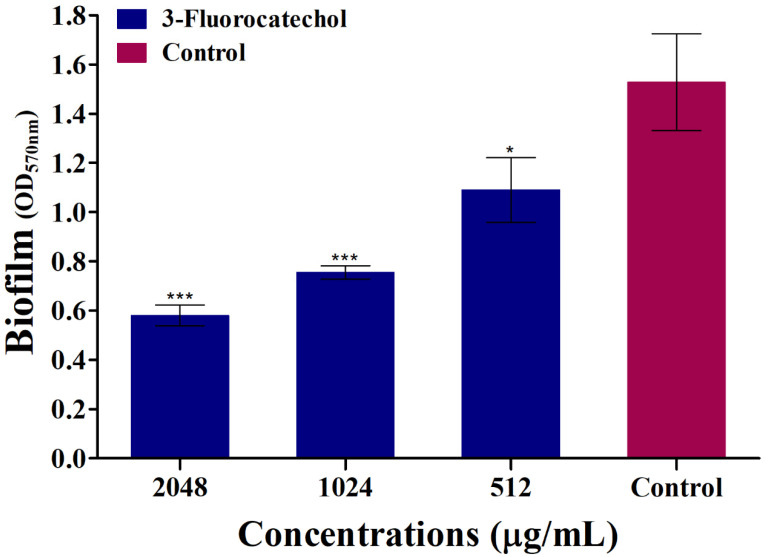
The effect of various concentrations of 3-FC on the eradication of pre-formed biofilm was assessed. Biofilm was quantified by measuring optical density at 570 nm after crystal violet staining. Asterisks indicate a statistically significant difference compared to the untreated control (* *p* < 0.05, *** *p* < 0.0001).

3-FC demonstrated concentration-dependent biofilm eradication activity against existing *S. aureus* biofilms. The highest dose of 2048 µg/mL had a significant eradication effect, eliminating pre-formed biofilms by 69.5% (Figure 4). Several potential compounds with concentration-dependent action have been developed through research on biofilm eradication agents against *S. aureus*. A previous report showed that bromophenazine derivatives effectively removed biofilms from *S. aureus* strains, including MRSA, with MBEC values ranging from 100 to 200 μM [51]. Similarly, vancomycin demonstrated a time-dependent eradication effect, eliminating biofilms at concentrations of 200 mg/L or greater after 28 days under static conditions [52]. Compared with its original drug, a fluorinated bisindole analogue demonstrated much higher levels of deleterious activity against established biofilms [20]. There was evidence that halicin was effective against mature biofilms aged 3 and 7 days, but an 8-fold increase in concentration was required [53].

### 2.4. Anti-Staphyloxanthin Activity of 3-FC

**Figure 5:**
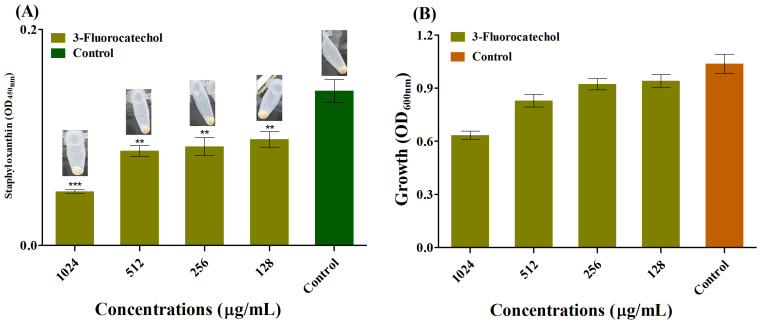
(**A**) Staphyloxanthin production was quantified by measuring optical density at 450 nm. Treatment with 3-FC inhibited staphyloxanthin production. (**B**) The optical density (OD_600_) was measured for data normalization. Asterisks indicate a statistically significant difference compared to the untreated control (** *p* < 0.001, *** *p* < 0.0001).

The anti-staphyloxanthin effect of 3-FC was quantified with the methanol extraction method. To account for differences in bacterial growth, pigment production was normalized by OD_600_ (Figure 5B). Treatment with 3-FC at concentrations ranging from 64 to 2048 µg/mL reduced pigment production compared with the untreated control. Inhibition of 32.6% was observed at 128 µg/mL, and this effect became progressively stronger with increasing concentrations, reaching 40.0% at 512 µg/mL and 65.9% at 1024 µg/mL (Figure 5A). This result can be compared with recent studies on other small-molecule inhibitors of staphyloxanthin [54]. In this study, ZY-214-4 (C19H11BrNO4) inhibited staphyloxanthin synthesis in several clinical *S. aureus* strains by 38.7% to 56.7% at a sub-inhibitory concentration of 4 µg/mL. Although higher concentrations of 3-FC are required to achieve a similar degree of pigment inhibition as ZY-214-4, 3-FC has a much simpler chemical structure, which may make it a more advantageous lead compound for further optimization.

### 2.5. Molecular Interactions of 3-Fluorocatechol with Virulence Proteins of S. aureus

**Figure 6:**
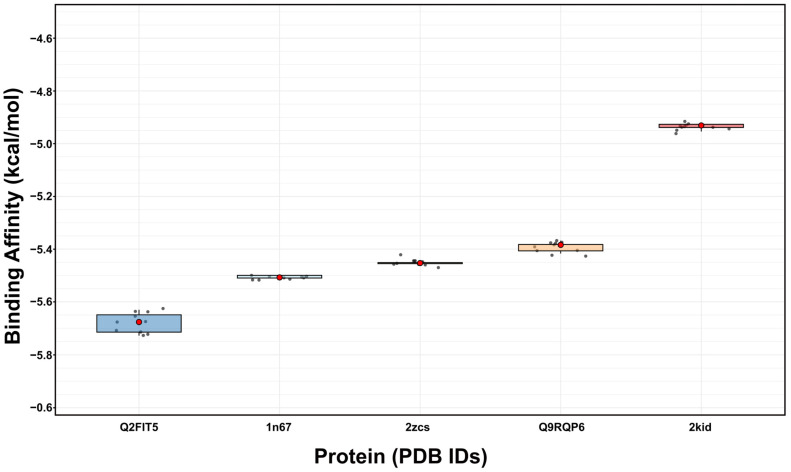
The binding affinity distribution of 3-FC (Y-axis, Kcal/mol) for *S. aureus* virulence proteins (X-axis) is visualized as a box plot.

**Figure 7:**
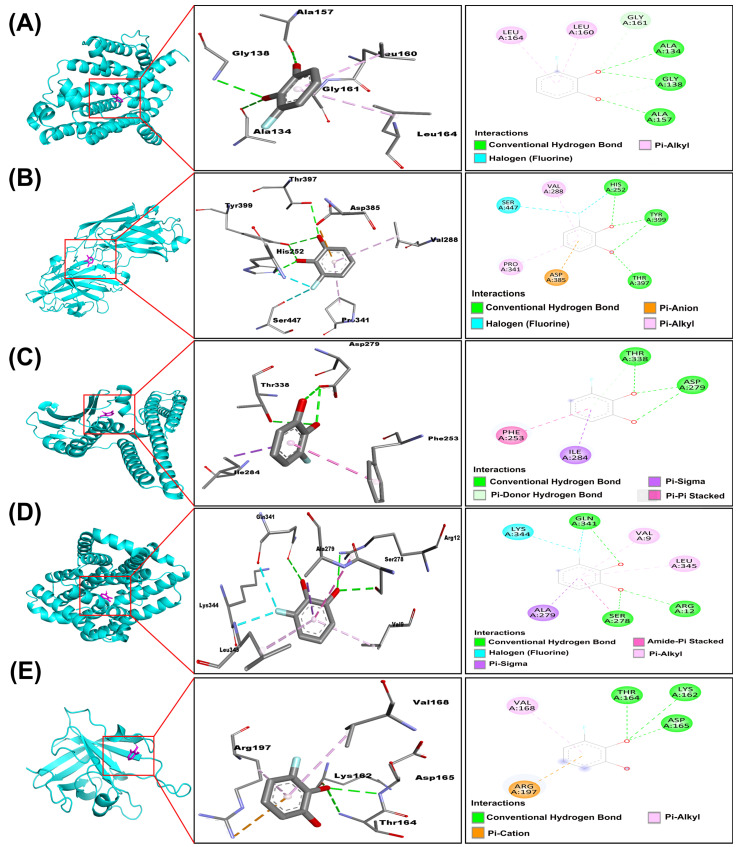
Predicted 3D and 2D interaction diagrams of 3-FC docked into active sites of *S. aureus* virulence proteins: (**A**) 2zcs, (**B**) 1n67, (**C**) Q2FIT5, (**D**) Q9RQP6, and (**E**) 2kid. Each part shows the overall protein–ligand complex structure (left), an enlarged view of the protein–ligand pocket (middle), and the 2D interaction diagram (right). Key interaction amino acid residues are labelled around the ligand. Different bonding types are indicated by distinct colors.

We particularly focused on five proteins (2zcs, 1n67, Q2FIT5, Q9RQP6, and 2kid) known to be essential for staphyloxanthin synthesis and biofilm formation (Table 2). Notably, 3-FC demonstrated strong binding affinities for these specific targets. Among them, Q2FIT5 showed the strongest binding affinity overall (−5.697 ± 0.027 kcal/mol). 1n67 (−5.505 ± 0.005) and 2zcs (−5.452 ± 0.007) also ranked among the top 5 strongest binders. Q9RQP6 (−5.392 ± 0.013) and 2kid (−4.933 ± 0.008) also showed significant binding, suggesting that 3-FC could effectively interfere with the staphyloxanthin and biofilm pathway (Figure 6). This binding is stabilized by multiple interactions, including hydrogen bonds, T-shaped π-π interactions, halogen bonds, π-anion interactions, π-σ interactions, and π-alkyl interactions. 2zcs formed three hydrogen bonds with Ala 134, Gly 138, and Ala 157, and two π-alkyl interactions with Leu 160 and 164 (Figure 7A). 1n67 formed three hydrogen bonds with His 252, Thr 397, Tyr 399, one halogen interaction with Ser 447, one π-anion interaction with Asp 385, and two π-alkyl interactions with Val 288, Pro 341 (Figure 7B). Q2FIT5 formed two hydrogen bonds with Asp 279, Thr 338, one π-σ interaction with Ile 284, and one π-π stacked interaction with Phe 253 (Figure 7C). Q9RQP6 formed three hydrogen bonds with Arg 12, Ser 278, Gln 341, one π-σ interaction with Ala 279, one halogen interaction with Lys 344, two π-alkyl interactions with Val 9, Leu 345 (Figure 7D). 2kid formed three hydrogen bonds with Lys 162, Thr 164, and Asp 165, one π-cation interaction with Arg 197, and one π-alkyl interaction with Val 168 (Figure 7E). According to the molecular interaction assay, 3-FC is suggested to inhibit the formation of staphyloxanthin and biofilms by binding to the active sites of the specific proteins mentioned above. Staphyloxanthin, the golden carotenoid pigment of *S. aureus*, serves as a crucial virulence factor that enhances bacterial resistance to oxidative stress and immune clearance [55,56]. Multiple studies have identified effective inhibitors targeting staphyloxanthin biosynthesis, particularly by inhibiting dehydrosqualene synthase (CrtM), the enzyme catalyzing the first committed step in staphyloxanthin production [56,57]. Phosphonoacetamide compounds exhibit potent CrtM inhibition, with nanomolar activity and effectiveness in whole cells and animal models [57]. Natural compounds also show promise, with farnesol competitively binding to CrtM and blocking staphyloxanthin synthesis [58].

### 2.6. ADMET Analysis of 3-Fluorocatechol

In silico ADMET of 3-FC was analyzed using pkCSM (https://biosig.lab.uq.edu.au/pkcsm/ (accessed on 17 November 2025)). 3-Fluorocatechol is predicted to have excellent intestinal absorption. Predicted human intestinal absorption was 90.182%. This is supported by the Caco-2 permeability value of 1.829 (log Papp in 10^−6^ cm/L), which is well above the high-permeability threshold (typically >0.9). The predicted water solubility is −1.239 (log mol/L), indicating high solubility. 3-Fluorocatechol was also predicted to be neither a substrate nor an inhibitor of P-glycoprotein. This result indicates a low probability of efflux from cells and of interacting with other compounds. However, 3-FC exhibited low skin permeability, indicating poor skin absorption (log Kp = −2.805). This analysis also predicts that 3-FC is effectively distributed into tissues. VDss is predicted to be 0.046 (log L/kg), suggesting distribution beyond the plasma compartment. The fraction unbound (FU) in human plasma is predicted to be 0.636, indicating that 63.6% of the drug would be free and pharmacologically active. On the other hand, 3-FC lacks CNS penetration, as indicated by both BBB permeability (log BB = −0.265) and CNS permeability (log PS = −2.116). 3-Fluorocatechol was supposed to be neither a substrate nor an inhibitor for enzymes (CYP2D6, CYP3A4, CYP1A2, CYP2C19, and CYP2C9). This suggests a low potential for metabolic drug–drug interactions. The total clearance of 3-FC was predicted to be low at 0.021 (log ml/min/kg). This in vivo half-life rate suggests a potentially long half-life. The compound was also predicted not to be a substrate for the renal organic cation transporter 2 (OCT2). The toxicity of 3-FC was also assessed. It was anticipated to be nontoxic in the AMES toxicity test. Critically, it was projected to be a non-inhibitor of both hERG I and II channels, indicating a low risk of cardiotoxicity. Furthermore, no hepatotoxicity or skin hypersensitivity was expected. The projected oral rat acute toxicity (LD50) was 1.785 mol/kg, while the minnow toxicity was 2.409 log mM, indicating a limited potential for acute toxicity.

## 3. Limitations of the Study

Despite the effective antibiofilm and antivirulence activity of 3-FC, several limitations make its complete application as a potential therapeutic drug difficult. The in silico docking studies in the present study show moderate binding energies (−4.9 to −5.7 kcal/mol) between 3-FC and the receptor, suggesting possible ligand–protein interactions; however, these findings cannot directly claim the actual inhibitory effects on virulence factors. Hence, it would be considered a preliminary finding until it is validated through multiple experimental approaches, such as gene expression analysis of the associated genes and enzymatic activity assays. Furthermore, ADMET-based pharmacokinetic analyses are considered in silico predictions that may also not hold up in experimental analysis. Although ADMET predictions indicate favorable intestinal absorption and low toxicity of 3-FC, these inferences remain speculative. This limitation is notable because the observed antibacterial effect of 3-FC against *S. aureus* required very high in vitro concentrations, raising concerns about its physiological potency and bioavailability. In addition, there is no experimental evidence on the 3-FC’s cytotoxicity, raising concerns about its safety profile. Hence, future studies are needed to validate the in silico docking interactions, conduct pharmacokinetic studies, perform comprehensive cytotoxicity assays, and examine structure–activity relationships.

## 4. Materials and Methods

### 4.1. Reagents, Pathogens, Culture Media, and Instruments

Compound 3-fluorocatechol (CAS RN: 363-52-0; purity > 98.0%) was purchased from Tokyo Chemicals Industry Co., Ltd., Japan. The microbial pathogens, including *Staphylococcus aureus* (KCTC 1916), *Pseudomonas aeruginosa* PAO1 (KCTC 1637), *Escherichia coli* (KCTC 1682), *Listeria monocytogenes* (KCTC 3569), *Streptococcus mutans* (KCCM 40105), *Klebsiella pneumoniae* (ATCC 4352), *Candida albicans* (KCCM 11282), and MRSA (KCCM 40510), were used in this study. For bacterial cultivation, tryptic soy broth (TSB) and tryptic soy agar (TSA) were used as growth media, except for *C. albicans*, which used potato dextrose broth (PDB). The optical density (OD) was measured using a microplate reader (Synergy HTX Multi-Mode Microplate Reader, BioTek Instruments, Canada).

### 4.2. Determination of Minimum Inhibitory Concentration (MIC)

The MIC was determined by broth microdilution [59]. A stock solution of 3-FC was prepared at 10 mg/mL in 1 mL of sterile distilled water. The MIC was determined using the serial dilution method. In 24-well microplates, two-fold serial dilutions of 3-FC were prepared to achieve final concentrations ranging from 2048 µg/mL to 64 µg/mL. Further, 300 µL was transferred to 96-well microplates in triplicate. Bacterial inoculum broth without 3-FC served as the untreated positive control. The plate was incubated at 37 °C for 24 h. The MIC was defined as the lowest concentration of 3-FC required to completely suppress visual growth. A microplate reader was used to measure the optical density (OD) at 600 nm. To assess the spectrum of 3-FC activity, this process was carried out simultaneously across multiple species, including *S. aureus*, *P. aeruginosa*, *E. coli*, *S. mutans*, *L. monocytogenes*, *K. pneumoniae*, MRSA, and *C. albicans*. All measurements were obtained from three repetitions.

### 4.3. Biofilm Inhibition Assay

The effect of 3-FC on biofilm development was measured with a crystal violet (CV) staining experiment [60,61]. An overnight seed culture was diluted 1:100 into fresh sterile TSB (OD_600_ = 0.05). Cells were treated with sub-MIC (3-FC) concentrations (64–2048 µg/mL) in a 24-well microplate and subsequently transferred to a 96-well microplate. The untreated positive control was inoculated with broth lacking 3-FC. The plate was incubated at 37 °C for 24 h without shaking. Following incubation, planktonic cells were removed, and the wells were cleaned three times with sterile phosphate-buffered saline (PBS). The remaining adhering biofilms were stained with 0.1% CV for 25 min. To remove excess stain, the wells were washed with water, and the attached color-stained cells were solubilized in 200 µL of 95% ethanol. The absorbance, which corresponds to the total biofilm mass, was measured at 570 nm. All measurements were repeated three times.

### 4.4. Quantification of Viable Biofilm Cells (CFU Enumeration)

To assess the viability of biofilm cells treated with 3-FC, colony-forming units (CFUs) were enumerated using previously described procedures [62]. *S. aureus* biofilms were generated on a 96-well plate for 24 h at 37 °C in static circumstances (no shaking) and in the presence of 3-FC at various sub-MIC concentrations (range from 64 µg/mL to 2048 µg/mL). Samples without 3-FC were used as untreated controls. Following incubation, the planktonic cells were gently removed, and the wells were rinsed three times with TSB to remove any weakly attached bacteria. To dislodge the biofilm, add 300 µL of TSB to each well and scrape the bottom with pipette tips. The obtained bacterial suspension was serially diluted 10-fold to 10^−4^. 100 µL of each dilution was added to TSA plates and incubated at 37 °C for 24 h. The viable bacterial counts of the biofilm cells were counted and expressed as Log10 CFU. All experiments were done in triplicate.

### 4.5. Eradication of Preformed Biofilm

The ability of 3-FC to eliminate existing biofilms was assessed using a previously reported approach [63]. Biofilms were generated by transferring 300 µL of a diluted overnight bacterial culture in TSB to a 96-well microplate and incubating for 24 h at 37 °C without shaking. Following the initial growth phase, planktonic cells were removed, and the wells were rinsed three times with PBS. Fresh sterile TSB with various dilutions of 3-FC (varying from 4 µg/mL to 2048 µg/mL) was applied to the wells containing the pre-formed biofilms. Sterile TSB was also added to the control group. The plate was incubated for an additional 24 h at 37 °C under static conditions. Following the removal of planktonic cells and washing, the remaining biofilm mass was determined using the CV staining method described in Section 4.3. All measurements were carried out in triplicate.

### 4.6. Examination of Biofilm Cells Under SEM

The seed cultures of *S. aureus* (OD_600_ = 0.05) were added to 24-well microplates containing a sterile nylon membrane (0.5 × 0.5 cm). The sub-MIC of 3-FC (2048 μg/mL) was also added into the well and incubated at 37 °C without shaking for 24 h to allow for biofilm formation. Inoculated broth without 3-FC served as an untreated control. After 24 h incubation, 60 µL of 2% formaldehyde and 2.5% glutaraldehyde were added to each well, and the mixture was incubated at 4 °C for 12 h to stabilize cell structure. After incubation, nylon membranes were carefully transferred to a new plate, and the planktonic bacteria were discarded. Each well was washed three times with Phosphate-Buffered Saline (PBS). After PBS, it was dehydrated through a graded ethanol series (25%, 50%, 70%, 80%, 90%, and 100%) for 15 min at each concentration. Finally, the samples were dried using a freeze dryer. Prepared samples were coated with gold in a vacuum sputter coater and examined under a scanning electron microscope VEGA II LSU (TESCAN, Brno, Czech Republic) [64].

### 4.7. Staphyloxanthin Assay

The effect of 3-FC on the suppression of staphyloxanthin production, the characteristic golden color of *S. aureus*, was examined using the methanol extraction method [65]. An overnight culture of *S. aureus* (OD_600_ = 0.05) was treated with different concentrations of 3-FC (64 to 2048 µg/mL) in a 24-well plate. A positive untreated control was also included. The plate was incubated at 37 °C for 24 h with shaking at 200 rpm to promote growth and pigment synthesis. After incubation, the total cell density in each well was measured at 600 nm (OD_600_) for standardization. The culture was centrifuged (8000 rpm, 10 min, 4 °C) to separate the bacterial cells. After the supernatant was removed, the pellet was washed once with sterile PBS. The pellet was resuspended in 1 mL of methanol, incubated in a water bath at 55 °C for 15 min, and then vortexed to lyse the cells. A yellowish supernatant was obtained by repeating centrifugation. The OD at 450 nm was measured after the supernatant was transferred to a new 96-well microplate.

### 4.8. Selection and Preparation of S. aureus Virulence Factor

Several well-characterized regulatory proteins that play roles in virulence, pathogenicity, biofilm formation, and QS signaling in *S. aureus* were used as receptors for molecular docking using 3-FC as the ligand. The tertiary structures of all the proteins (Table 3) were obtained from the RCSB Protein Data Bank (https://www.rcsb.org). Heteroatoms and water molecules were removed from the protein structures. To improve structural stability, the resulting proteins were subjected to energy minimization using Swiss-PDB Viewer (SPDBV 4.10) [66]. The energy-minimized proteins were further processed using AutoDock Tool 1.5.6, in which polar hydrogens were added, Kollman charges were assigned, and atom types were configured to AD4 [67].

### 4.9. Preparation of 3-FC Ligand

The molecular structure of 3-FC was retrieved from PubChem and pre-processed for docking using Open Babel Software v3.1.1. The energy was minimized using the GAFF force field, and Gasteiger charges were assigned to each compound [75].

### 4.10. Molecular Docking Analysis of 3-FC with Diverse Virulence Factors of S. aureus

A well-established molecular docking approach was employed using AutoDock Vina v1.2.0, after generating PDBQT files for all proteins and ligands [76]. The grid box was positioned based on the previously reported active site coordinates, and its size was set to 40 Å with a 0.5 Å spacing. The coordinates of key catalytic residues were extracted from UniProt entries and averaged to represent the grid center. To ensure consistency and variability in docking results, each protein–ligand complex was subjected to 10 independent docking runs using a consistent random seed. The binding free energy (kcal/mol) was presented as the mean ± 95% CI (confidence interval). The docking results were evaluated based on the lowest binding energy (kcal/mol) and the most favorable ligand binding poses to create the final docked complexes. The molecular interactions of the protein–ligand complex were further analyzed using BIOVIA Discovery Studio 2021 (BIOVIA Discovery Studio|Dassault Systèmes).

### 4.11. Statistical Analysis

Data were analyzed using a one-way ANOVA followed by Dunnett’s multiple-comparison test for statistical significance (*p*-value) in GraphPad Prism 7.0 (GraphPad Software Inc., San Diego, CA, USA), and plotted as means ± SD. Significance was indicated by *** *p* < 0.0001, ** *p* < 0.01, and * *p* < 0.05.

## 5. Conclusions

The present study reassesses the additional antibacterial effect of 3-FC, which was earlier found as a suicide inhibitor of catechol 2,3-dioxygenase in *P. putida*. 3-FC has antibiofilm and antivirulence activities against *S. aureus*. Although 3-FC did not exhibit significant bactericidal activity against *S. aureus*, it effectively prevents biofilm formation at sub-MIC levels. Therefore, future studies are needed to evaluate the application of 3-FC as an adjuvant, particularly to determine whether it can exhibit synergistic effects with conventional antibiotics against *S. aureus*, thereby allowing a reduction in the effective dose of 3-FC. The impact on the eradication of established mature *S. aureus* biofilms was also discovered to be effective in the presence of a high concentration of 3-FC. As a result, it can suppress the initial stage of biofilm formation while eradicating mature *S. aureus* biofilms. Furthermore, 3-FC dramatically reduced staphyloxanthin formation, a recognized antioxidant pigment that contributes to bacterial resistance to oxidative stress, suggesting potent anti-virulence activity. The molecular docking analysis demonstrates that 3-FC significantly binds to proteins involved in QS signaling, adhesion, pigment production, and cell wall anchorage in *S. aureus*, thereby limiting the pathogen’s capacity to invade and evade host defenses. Furthermore, the ADMET study confirms that molecule 3-FC has favorable pharmacokinetic properties. However, it should be noted that the observed effects required relatively high concentrations. Therefore, 3-FC is proposed as a promising structural scaffold for the development of novel antibacterial and antivirulence agents. Future research should focus on structural optimization to enhance potency at lower doses and on validating efficacy and safety in in vivo models.

## Figures and Tables

**Table 1:** MIC values of 3-fluorocatechol towards diverse microbial pathogens.

Strains	(MIC μg/mL)
*S. mutans* (KCCM 40105)	>2048 µg/mL
*S. aureus* (KCTC 1916)	>2048 µg/mL
*E. coli* (KCTC 1682)	>2048 µg/mL
*L. monocytogenes* (KCTC 3569)	>2048 µg/mL
*K. pneumoniae* (ATCC 4352)	>2048 µg/mL
Methicillin-resistant *S. aureus* (MRSA; KCCM 40510)	>2048 µg/mL
*P. aeruginosa* PAO1 (KCTC 1637)	512 µg/mL
*C. albicans* (KCCM 11282)	512 µg/mL

KCTC (Korean Collection for Type Cultures); KCCM (Korean Culture Center of Microorganisms); ATCC (American Type Culture Collection).

**Table 2:** The binding affinities (±95% confidence intervals) and detailed docking results of 3-fluorocatechol against several virulence factors of *S. aureus*.

Target Proteins		3-FC		
Protein ID	Name	Mean Affinity ± 95%CI	Binding Residues	Interaction Types
2zcs	Carotenoid dehydrosqualene synthase	−5.452 ± 0.007	Ala 134, Gly 138, Ala 157, Leu 160, Gly 161, Leu 164	Conventional hydrogen bond, Carbon–hydrogen bond, Pi-Alkyl
1n67	Clumping Factor A	−5.505 ± 0.005	His 252, Val 288, Pro 341, Asp 385, Thr 397, Tyr 399, Ser 447	Conventional hydrogen bond, Halogen, Pi-Anion, Pi-Alkyl
Q2FIT5	Histidine protein kinase SaeS	−5.697 ± 0.027	Phe 253, Asp 279, Ile 284, Thr 338	Conventional hydrogen bond, Pi-Donor hydrogen bond, Pi-Sigma, Pi-Pi
Q9RQP6	Poly-beta-1,6-N-acetyl-D-glucosamine export protein	−5.392 ± 0.013	Val 9, Arg 12, Ser 278, Ala 279, Gln 341, Lys 344, Leu 345	Conventional hydrogen bond, Amide-Pi, Halogen, Pi-Alkyl, Pi-Sigma
2kid	Sortase A	−4.933 ± 0.008	Lys 162, Thr 162, Asp 165, Val 168, Arg 197	Conventional hydrogen bond, Pi-Alkyl, Pi-Cation

**Table 3:** The biofilm, QS, and virulence-associated protein from *S. aureus*.

Protein	PDB and Uniport ID	Functional Roles	References
CrtM	2zcs	Staphyloxanthin synthesis	[56]
ClfA	1n67	Facilitates bacterial adhesion to fibrinogen and endothelial cells.	[68]
IcaD FmtA	Q9RQP6	Stabilizes IcaA activity and promotes PIA synthesis.	[69,70,71,72]
SaeS	AF-Q2FIT5-F1	Part of the SaeRS two-component system regulating immune evasion and biofilm genes.	[73]
Sortase A	2kid	Anchors surface proteins to peptidoglycan, promoting colonization and biofilm formation.	[74]

## Data Availability

The data presented in this study are available on request from the corresponding author.

## Abbreviations

3-FC, 3-fluorocatechol; MIC, Minimum Inhibitory Concentration; PBS, phosphate-buffered saline; SEM, scanning electron microscopy; TSB, tryptic soy broth: CV, crystal violet; TSA, tryptic soy agar; ADMET; Absorption Distribution Metabolism Excretion Toxicity.
